# Life cycle inventory and life cycle impact assessment datasets of PDO Feta production in Stymfalia region, Greece

**DOI:** 10.1016/j.dib.2023.109207

**Published:** 2023-05-07

**Authors:** Samuel Le Féon, Andreas Papadakis, Gwenola Yannou-Le Bris, Julie Auberger, Dimitrios Chatzitheodorou, Joël Aubin, Caroline Pénicaud

**Affiliations:** a Université Paris-Saclay, INRAE, AgroParisTech, UMR SayFood, 91120, Palaiseau, France; b Synelixis SA, 10 Farmakidou, 34100, Chalkida, Greece; c UMR SAS, INRAE, Institut Agro, 65 rue de Saint Brieuc, 35042, Rennes, France; d Stymfalia SA, Kalliani Korinthia, Adresscode 200 16, Greece

**Keywords:** Environmental assessment, LCA, Cheese production, Data inventory

## Abstract

Considering and reducing the environmental impacts has become one of the main concerns of agri-food systems. More specifically, the agri-food sector is increasingly confronted to the necessity of quantifying environmental impacts, e.g., to eco-design their products or to inform the consumers. Literature shows a high variability in environmental impacts between existing systems, as for example between cheeses and the necessity of more case studies to validate statements. In this context, this data paper provides some data related to Feta production in Greece, based on 8 farms of a cooperative (7 sheep livestock and one goat livestock). Feta cheese is PDO (Protected Designation of Origin), composed solely of goat's milk and sheep's milk under specific percentages (at least 70% sheep). More specifically, the data paper displays all the data used to obtain environmental impacts (calculated by using life cycle assessment (LCA)) of the production of Feta, from cradle to consumer. It includes the – sheep and goat – milk productions, the transformation into cheese, the packaging and the transport to wholesalers, then stores and then consumers. The raw data have mostly been obtained through interviews and surveys with the cheese and milk producers and complemented by literature. Data were used to build a life cycle inventory (LCI). For the milk production, the LCI was modeled using MEANS InOut software. For the whole LCI, Agribalyse 3.0 and Ecoinvent 3.8 were used as background databases, with modifications to reflect Greek context. The dataset also compiles the life cycle impact assessment (LCIA). The characterization method used is method EF3.0. This dataset participates in filling two gaps: (1) providing data to represent the variability between Feta cheese production systems and (2) providing data linking impacts of farm, transformation, retail and transport in a value chain perspective. This is done by (1) enlarging the perimeter when most studies found in literature focus on one stage (e.g. the production of milk) and (2) applying LCA to data specific to a regional production (Stymfalia in Greece).

**Specifications Table**

**Table:** 

Subject	Environmental engineering
Specific subject area	Environmental assessment of agri-food value chains
Type of data	Table CSV
How the data were acquired	The raw data have mostly been obtained through interviews and surveys with the cheese and milk producers. The collection was realized by a representative of the local dairy based on a template provided by the LCA team. They were supplemented by scientific and technical literature as well as model calculations (MEANS-InOut software). Background data are issued from AGRIBALYSE 3.0 and Ecoinvent 3.8 databases. Life-cycle impact assessment was done using Simapro 8.4 and the “EF 3.0 Method”.
Data format	Raw Analyzed
Description of data collection	The raw data have mostly been obtained through interviews and surveys with the cheese and milk producers. The collection was realized by a representative of the local dairy based on a template provided by the LCA team. The dataset was complemented by literature. Ecoinvent 3.8 and Agribalyse 3.0 were used for background data. LCIA data were calculated using the software Simapro and the characterization method “EF 3.0 method (adapted)”
Data source location	Foreground data collected at farm and dairy • Institution: Stymfalia SA • City/Town/Region: Kaliani • Country: Greece Agribalyse 3.0 • Institution: ADEME • City/Town/Region: Nantes • Country: France • https://agribalyse.ademe.fr/
	Ecoinvent 3.8 • Institution: Ecoinvent • City/Town/Region: Zurich • Country: Switzerland • https://ecoinvent.org/ MEANS-InOut • Institution: INRAE and CIRAD • City/Town/Region: Rennes • Country: France • https://www6.inrae.fr/means
Data accessibility	Repository name: Recherche Data Gouv - INRAE Dataverse Data identification number: https://doi.org/10.57745/1 × 4QYO Direct URL to data: https://doi.org/10.57745/1 × 4QYO

## Value of the Data


 

• The article presents a unique set of LCI and LCIA data for on the production of Feta in Stymfalia region, Greece.
• Data from different farms are used and variability between farms is presented.
• The LCI for sheep and goat milks were calculated using MEANS InOut software.
• The article can be used to identify hotspots for Feta production as well as for sheep and goat milk productions on a regional scale.

## 1 Objective

Literature shows a high variability in environmental impacts between existing systems, as for example between cheeses [1] and the necessity of more case studies to validate statements [2]. In this context, this data paper provides some data related to Feta production in Greece, based on 8 farms of a cooperative (7 sheep livestock and one goat livestock). This work was conducted in the framework of EU FAIRCHAIN project Feta cheese is PDO (Protected Designation of Origin), composed solely of goat's milk and sheep's milk under specific percentages (at least 70% sheep). More specifically, the data paper displays all the data used to obtain environmental impacts (calculated by using life cycle assessment (LCA)) of the production of Feta, from cradle to consumer. This dataset has the objective to participate in filling two gaps: (1) providing data to represent the variability between Feta cheese production systems and (2) providing data linking impacts of farm, transformation, retail and transport in a value chain perspective.

## 2 Data Description

The dataset associated with this article contains eight files with information on raw data, inventory data and the LCIA:

1. (FAIRCHAIN)CS-Gre_raw_data_milk_production: it describes the raw data collected with the dairy to build the inventories and completed by literature for the milk productions. Type of data (collected, literature, calculated) is specified as well as hypothesis.
2. (FAIRCHAIN)CS-GRE_raw_data_other_stages: it describes the raw data collected with the dairy to build the inventories and completed by literature for other stages (processing, packaging, distribution). Type of data (collected, literature, calculated) is specified as well as hypothesis.
3. (FAIRCHAIN)CS-Gre_LCI_milk_production: it describes the life cycle inventories calculated with MEANS InOut and adapted with Simapro. The values of input/output flows are specified as well as the database and processes used to model them.
4. (FAIRCHAIN)CS-Gre_LCI_one_year_production: it describes the life cycle inventory for one-year of production of the cheese factory. The values of input/output flows are specified as well as the database and processes used to model them.
5. (FAIRCHAIN)CS-Gre_LCI_packaging: it gives details on the simplified inventories used to model the packaging solutions.
6. (FAIRCHAIN)CS-Gre_LCIA_milk_production_contributions: it presents LCIA at farm for the production of all milks involved as well as for the weighted average for sheep milk.
7. (FAIRCHAIN)CS-Gre_LCIA_one_year_value_chain: it presents LCIA for the whole value chain for one-year production.
8. Bibliography: it details the additional bibliographical sources cited in the different data files.

## 3 Experimental Design, Materials and Methods

### 3.1 LCA methodology

Our work followed ISO 14040 [3].

#### 3.1.1 Goal and scope

In the framework of FAIRCHAIN, the LCA is a first step analysis towards the implementation of innovations. The objectives are to collect data to build a LCI that is representative of the current situation. The calculation of the environmental impacts (LCIA) is carried out with the main objective to constitute the frame of reference that will allow to evaluate the environmental impacts of the innovation in the future. The innovation should only have a marginal effect on markets. Following these objectives, an attributional approach is chosen.

#### 3.1.2 System boundaries

**Fig. 1:**
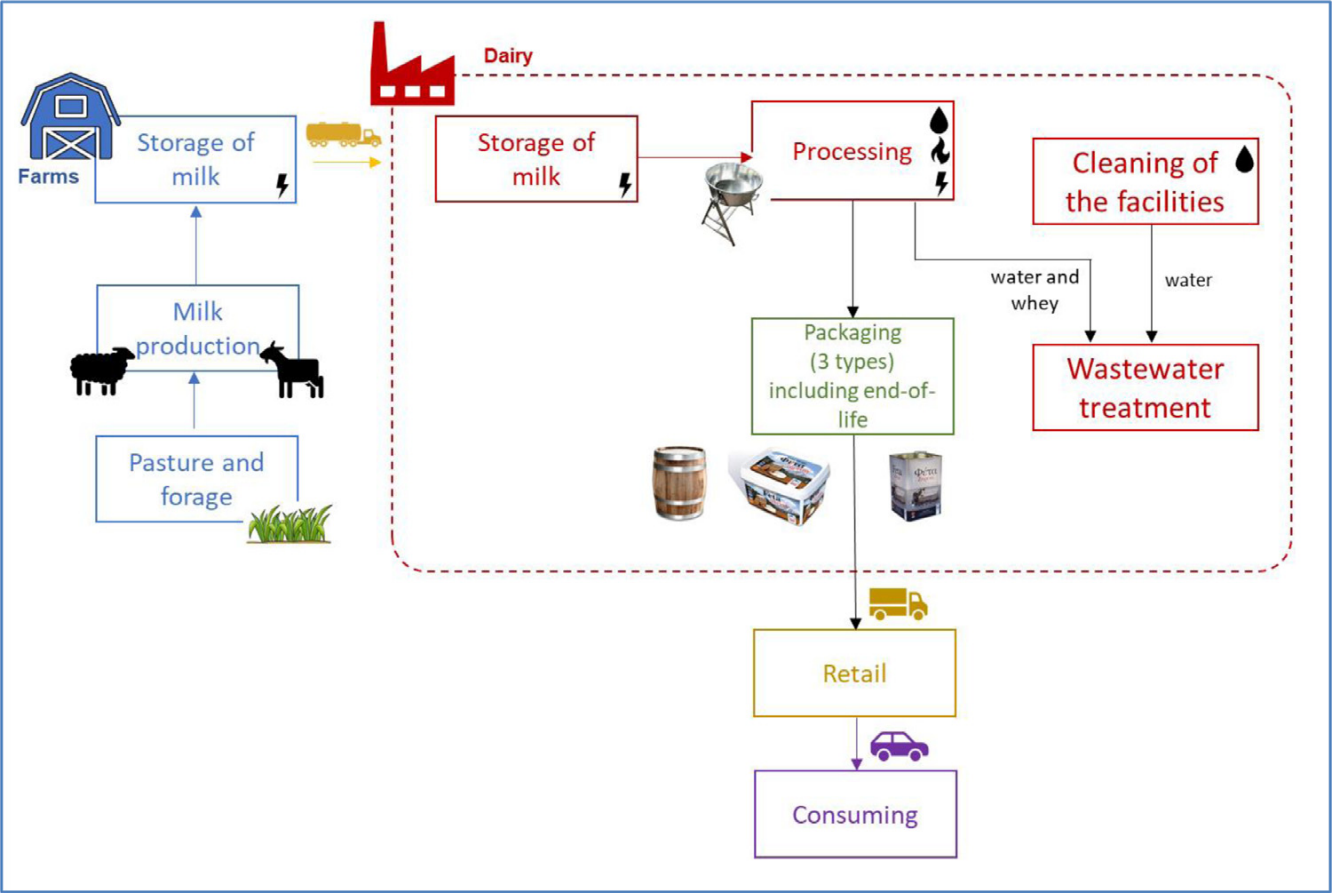
Simplified flow diagram of the studied system from cradle to grave

The system boundaries include (Fig. 1):

- The sheep and goat milk production from 8 farms;
- The cold storage of milk at farms before collection;
- The collection and transport of the milk to the cheese factory;
- The production of Feta cheese. Greek yogurt and myzithra are also produced in the factory but they are not considered in the scope of the assessment;
- The cleaning of the cheese factory;
- The wastewater treatment of water used at the cheese factory;
- The management of the whey: the totality of the whey is considered sent to wastewater treatment;
- The packaging of the Feta, including four different packaging types with end-of-life treatments;
- The transports to wholesalers then to stores;
- The consumption.

The processes are described in [4].

#### 3.1.3 Functional unit

The functional units are defined as “the annual production of Feta at the cheese factory”. However, LCIA is also calculated and provided at farms level with functional units defined as “1 kg of milk produced in farm [X]”.

#### 3.1.4 Life cycle inventory

##### 3.1.4.1 At farm

The Greek CS concerns sheep and goat milk cheeses produced in Stymfalia region, in Peloponnese. Specific data were provided by the dairy cooperative for 7 sheep farms and 1 goat farm. These data were used to model and calculate LCI for each farm using MEANS-InOut software (https://www6.inrae.fr/means). The Agribalyse 2022 model set was selected to calculate the emissions. The models associated are named in dataset_models_MEANS. The LCI generated were exported in Simapro software then adapted to the context. Firstly, the electricity mix was modified from French (data set by default in MEANS-InOut) to Greek. Secondly, the electricity consumption related to the cold storage of milk is separated from the rest of electricity consumption (as the information was available and should be valuable for the data users). The related dataset is (FAIRCHAIN)CS-Gre_raw_data_milk_production.tab.

##### 3.1.4.2 Transformation

The dataset at the cheese factory represents one-year production, involving more milk than produced by the 8 farms for which specific data were available ((FAIRCHAIN)CS-Gre_LCI_one_year_production). Following, the judgement of the dairy representative about the representativity of the sample, the goat's milk is taken as proxy for goat's milk and an average sheep milk has been considered as input for the cheese factory, based on a weighted average of the 7 sheep's milks.

$$Inventory_{1\;kg\;average\;sheep\;milk}=\;\sum_{i}Inventory_{1\;kg\;of\;sheep\;milk\;from\;farm\;i}*\frac{Q_{i}}{Q_{T}}$$

Where Q_i_ is the quantity of milk produced at farm i and Q_T_ is the total quantity of sheep milk produced in the 7 studied farms. The collection and transportation of the milk from farms to the cheese factory is modelled in the same way, based on the weighted average distance from farms to the cheese factory.

Other data at the cheese factory are based on specific data and include:

- The storage at factory, including tanks and energy;
- The processing, including ingredients (salt, rennet and lactic bacteria), energy (electricity and heat) and water;
- The cleaning, including water, energy and detergents (nitric acid and sodium hydroxide);
- The wastewater treatment of water and whey.

##### 3.1.4.3 Packaging, distribution and consumption

As this part of the inventory was not part of the perimeter under FAIRCHAIN project, it should be only considered as a first approach aiming at representing being included in the whole value chain. However, it provides valuable inputs from the cheese factory and has been judged relevant to be included in the dataset.

Four types of packaging and their respective shares in the whole cheese packaging were modelled based on specific data provided by the cheese factory: two tin containers, one wood barrel and one plastic container((FAIRCHAIN)CS-Gre_LCI_packaging.tab). The repartition between those packaging is given in (FAIRCHAIN)CS-Gre_LCI_one_year_production. In the absence of specific data, the packages were considered managed following generic mixes available in Ecoinvent database (FAIRCHAIN)CS-Gre_LCI_packaging.tab).

The transport to wholesalers was extrapolated based on the three biggest wholesalers (that represent 25% of the total volume). In the same way, the transport to stores was modelled based on the three biggest volumes delivered by the biggest wholesaler (100% of the total volume of the wholesaler). This method has been validated by the cheese factory as representative of its market.

Finally, the transport to consumer and the storage at home were modelled to consider the whole value chain. As no specific data were available, this is based on generic literature data and subject to high uncertainty.

##### 3.1.4.4 Databases

Agribalyse 3.0 and Ecoinvent 3.8 databases are used for background data.

#### 3.1.5 Life cycle impact assessment

The LCIA is realized using Simapro 8.3 and EF 3.0 as characterization method. It is compiled into two separated files:

- (FAIRCHAIN)CS-Gre_LCIA_milk_production_contributions: it presents LCIA at farm for the production of all milks involved as well as for the weighted average for sheep milk. For each farm, for each indicator, the contributions are given for direct emissions, feedstock (wheat straw, grass, barley and water), the building, the electricity consumption, the water used for cleaning and the litter (wheat straw).
- (FAIRCHAIN)CS-Gre_LCIA_one_year_value_chain: it presents LCIA for the whole value chain for one-year production. For each indicator, contributions are details by subprocesses. They are given in absolute value, related to the unit of the indicator (top table) and relatively to the total impact (bottom table).

## Ethics Statements

This work did not involve human subjects or laboratory animal, therefore did not meet any ethical issues.

## CRediT authorship contribution statement

**Samuel Le Féon:** Conceptualization, Methodology, Investigation, Software, Writing – original draft. **Andreas Papadakis:** Conceptualization, Writing – review & editing, Data curation. **Gwenola Yannou-Le Bris:** Conceptualization, Writing – review & editing. **Julie Auberger:** Software, Conceptualization, Writing – review & editing. **Dimitrios Chatzitheodorou:** Data curation, Writing – review & editing. **Joël Aubin:** Conceptualization, Writing – review & editing. **Caroline Pénicaud:** Conceptualization, Writing – review & editing, Supervision.

## Declaration of Competing Interest

The authors declare that they have no known competing financial interests or personal relationships that could have appeared to influence the work reported in this paper.

## Data Availability

(FAIRCHAIN)CS-Gre_datapaper (Original data) (entrepot.recherche.data.gouv.f)
